# Early Pup Removal Leads to Social Dysfunction and Dopamine Deficit in Late Postpartum Rats: Prevention by Social Support

**DOI:** 10.3389/fgwh.2021.694808

**Published:** 2021-07-08

**Authors:** Millie Rincón-Cortés, Anthony A. Grace

**Affiliations:** 1 Department of Neuroscience, University of Pittsburgh, Pittsburgh, PA, United States,; 2 Department of Psychiatry, University of Pittsburgh, Pittsburgh, PA, United States,; 3 Department of Psychology, University of Pittsburgh, Pittsburgh, PA, United States

**Keywords:** pup removal, postpartum, maternal, social, dopamine, ventral tegmental area, depression

## Abstract

Offspring interaction is among the most highly motivated behaviors in maternal mammals and is mediated by mesolimbic dopamine (DA) system activation. Disruption or loss of significant social relationships is among the strongest individual predictors of affective dysregulation and depression onset in humans. However, little is known regarding the effects of disrupted mother–infant attachment (pup removal) in rat dams. Here, we tested the effects of permanent pup removal in rat dams, which were assigned to one of three groups on postpartum day (PD) 1: pups; pups removed, single-housed; or pups removed, co-housed with another dam who also had pups removed; and underwent a behavioral test battery during PD 21–23. *In vivo* electrophysiological recordings of ventral tegmental area (VTA) DA neurons were performed on PD 22 and 23 in a subset of animals. Pup removal did not impact sucrose consumption or anxiety-like behavior, but increased passive forced swim test (FST) coping responses. Pup-removal effects on social behavior and VTA activity were sensitive to social buffering: only single-housed dams exhibited reduced social motivation and decreased numbers of active DA neurons. Dams that had pups removed and were co-housed did not exhibit changes in social behavior or VTA function. Moreover, no changes in social behavior, FST coping, or VTA activity were found in socially isolated adult virgin females, indicating that effects observed in dams are specific to pup loss. This study showed that deprivation of species-expected social relationships (pups) during the postpartum precipitates an enduring negative affect state (enhanced passive coping, blunted social motivation) and attenuated VTA DA function in the dam, and that a subset of these effects is partially ameliorated through social buffering.

## INTRODUCTION

The mother–infant attachment bond is among the strongest social attachments formed by mammals (1). Mother–infant attachment is an adaptive and reciprocal process consisting of dynamic and complex behavioral and physiological interactions, which are facilitated by sensory and thermotactile cues embedded within the mother–infant dyad (2, 3). Disruption and/or removal of these processes may negatively affect both members in the dyad (4). Perturbations in mother–infant attachment induce immediate neurobiological changes in the offspring that shape subsequent development, lead to neurobehavioral dysregulation and increased vulnerability to psychopathology during later life (5, 6). Mothers experiencing infant loss exhibit long-lasting, elevated levels of anxiety and depression compared with mothers with a live born child (7). Since human depression often entails loss of or disruption of significant social relationships (8, 9), animal models based on depression-like responses to disrupted mother–infant attachment are useful to study neural correlates associated with and/or contributing to these outcomes. However, less is known about the consequences of disrupting mother–infant attachment in the rat dam.

Although most rodent studies examining the effects of pup separation have largely focused on the offspring, several studies have emphasized the behavioral effects in the dam (10). In rats, brief mother–offspring separations (15 min × day, postpartum day (PD) 2–14) increase maternal behaviors toward pups, whereas long separations (3 h × day, PD 2–14; 5 h day, PD 1–17 or PD 1–21) decrease maternal behavior (11–13), alter maternal aggression and anxiety-like behavior (11, 14) and increase immobility in the forced swim test (FST) (14, 15). Similar to long-term pup separations, permanent pup removal shortly after birth also increases FST immobility in the dam even when tested 4 weeks later, and also impairs working memory/cognitive flexibility during the postpartum period, suggesting coexisting motivational and cognitive deficits (16–18). Notably, the behavioral alterations observed in dams following long-term separations or pup removal resemble those observed in stress-based animal models relevant to depression characterized by the dopamine(DA) system malfunction (19, 20). Indeed, increased expression of depression-related phenotypes is associated with downregulation (i.e., hypoactivity) of mesolimbic DA system function, specifically within ventral tegmental area (VTA) DA neurons (20–22). Importantly, significant shifts occur within this system to help the dam adapt and cope with her new life role as a mother (23–25). Within this context, pup presence and contact are highly rewarding to the dam and, as such, stimulate DA release and increase reward-related activity within the dam’s mesolimbic DA system (25, 26). We hypothesized that removing this source of motivationally salient input (i.e., pups) likely has adverse effects on mesolimbic DA function in the dam. However, no studies have examined DA system changes, particularly within the VTA, in response to deprivation of species-expected social stimuli (i.e., pup presence) during the postpartum period.

In women, increased feelings of attachment to their infant are associated with increased positive mood during the postpartum (27, 28). In rats, increased licking of pups is associated with decreased depressive-like behavior in the dam (15). Thus, the nature and duration of mother–infant attachment is an important determinant of maternal mood. Based on these data, we hypothesized that pup removal would induce long-lasting changes in maternal affect (i.e., increased negative coping, blunted social behavior) and VTA activity (i.e., reduced DA activity). Given that the presence of a conspecific can ameliorate stress effects on behavior and brain in humans and rodents such that organisms show better recovery from distress (i.e., social buffering) (29, 30), we also tested whether the neurobiological sequelae of the dam following pup removal was sensitive to social buffering. To this end, dams were assigned to one of three conditions on PD 1: pups present (Pups), pups removed and dam isolated (No Pups-ISO), pups removed and dam pair-housed (No Pups-PH). Animals remained in their assigned conditions until undergoing a behavioral test battery for anxiety- and depression-related phenotypes from PD 21 to 23 followed by *in vivo* electrophysiological recordings within the VTA.

## MATERIALS AND METHODS

### Animals

Timed-pregnant (gestational day 13) adult female Sprague–Dawley rats were shipped overnight (Envigo, Indianapolis, IN, United States) and arrived at our facility the next morning. Rats were housed individually in a temperature-controlled room on a 12-h light/dark cycle with food and water available *ad libitum*. The day each litter (eight pups minimum) was born was designated as PD 0. Within 24 h of birth (PD 1), the litters were culled to five male and five female pups for dams assigned to the Pups group (total *n* = 16), in order to be consistent with the only published study regarding the effects of pup removal on the dam’s behavior (17), and all pups were removed from the No Pups groups: pups removed and single-housed (No Pups-ISO, total *n* = 17), or pups removed and dam pair-housed with another dam that had pups removed (No Pups-PH, *n* = 19). All animals were assigned randomly to their condition. Animals of the same condition (No Pups-PH) were housed together to control for potential confounds due to different reproductive states. The estrous cycle was not monitored in dams since the postpartum period is characterized by persistent diestrus and cessation of ovarian cycling (31, 32).

As an additional control, separate cohorts of adult virgin females were subjected to a 3-week isolation period (21–23 days, to mimic the number of days the dams used in the experiments above) followed by behavioral testing or electrophysiological recordings to determine whether similar isolation effects are observed across reproductive conditions and to ensure that the effects observed in dams following pup removal are specific to pup loss and not social isolation (Virgin-PH: total *n* = 12, Virgin-ISO: total *n* = 11). All animals were kept undisturbed except for routine weekly animal care by the experimenter. The estrous cycle was not monitored in these animals given prior reports indicating no impact of estrous cycle in the FST immobility duration (33, 34) or in the three-chambered social approach test (35), as well as to avoid the confound of handling stress required for daily vaginal swabs. All experiments were performed in accordance with the guidelines outlined in the National Institutes of Health Guide for Care and Use of Laboratory Animals and approved by the Institutional Animal Care and Use Committee of the University of Pittsburgh.

### Behavioral Testing

**FIGURE 1 |:**
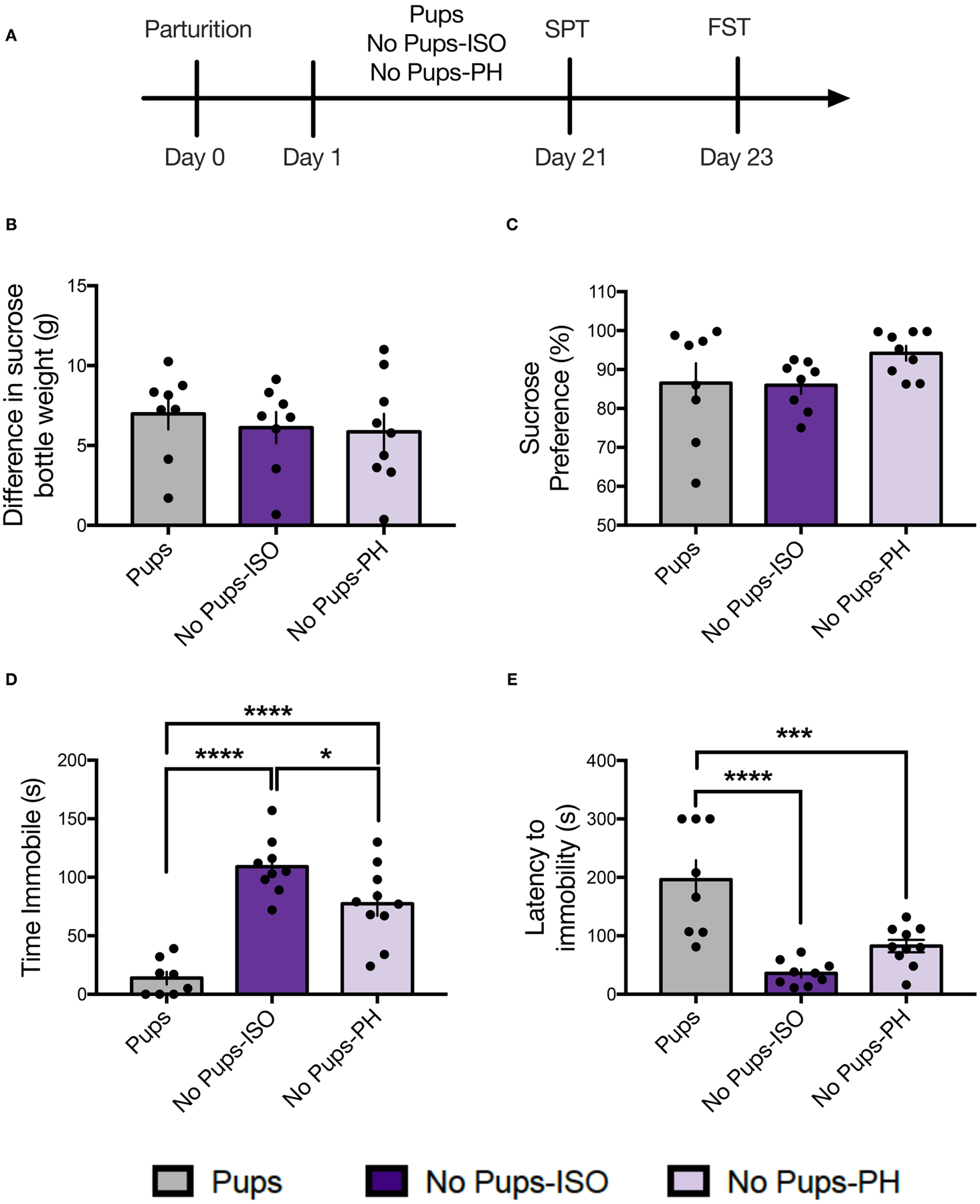
Pup removal did not impact maternal sucrose consumption or preference but increased passive coping in the forced swim test (FST) during the late postpartum period. **(A)** Schematic of the experimental timeline. **(B,C)** No effect of pup removal was found for **(B)** difference in sucrose bottle weight (*p* = 0.73) or **(C)** percentage of sucrose consumed (*p* = 0.16) in the sucrose preference test (SPT). **(D,E)** A main effect or pup removal was detected for **(D)** immobility duration and **(E)** latency to immobility (one-way ANOVA; *p* < 0.05, *n* = 8–10 per group,) in the FST. Both groups of dams that underwent early pup removal [No pups-isolated (ISO), No pups- pair-housed (PH)] exhibited **(D)** increased immobility and **(E)** reduced latency to immobility during the FST compared with the dams with pups (Tukey’s; *p* < 0.05). Error bars represent mean ± SEM. Gray bars represent control dams (Pups), dark purple bars represent dams that underwent pup removal and postpartum isolation (No Pups-ISO), and light purple represents dams that underwent pup removal but were co-housed (No Pups-PH). *p < 0.05, ***p < 0.001, ****p < 0.0001.

**FIGURE 2 |:**
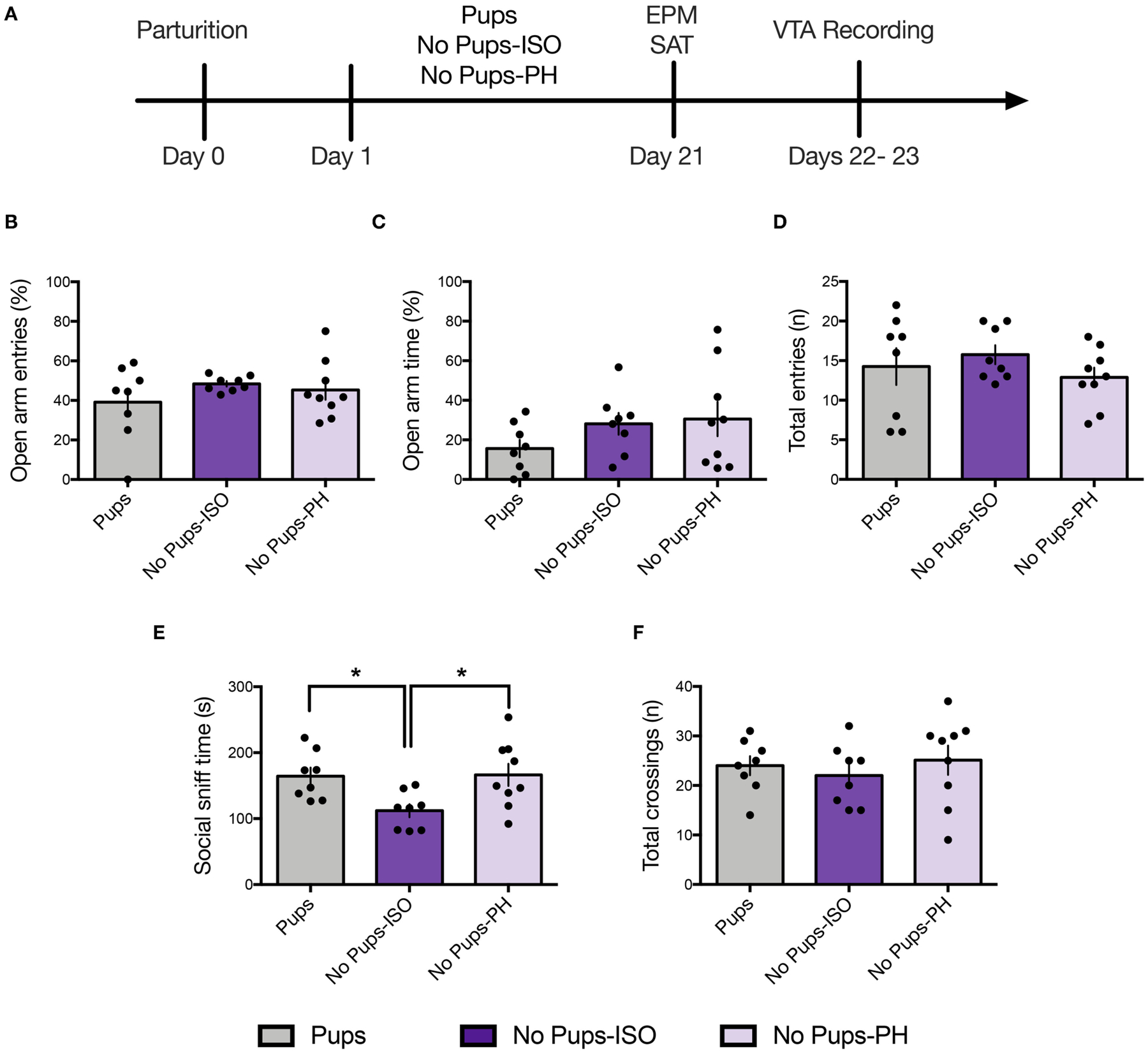
Pup removal spared anxiety-like behavior but reduced social motivation only in dams that were isolated during the postpartum period. **(A)** Schematic of the experimental timeline. **(B–D)** No effect of pup removal was found for anxiety-like behavior or locomotor activity in the elevated plus maze (EPM) (one-way ANOVA; 8–9 per group). All dams made **(B)** comparable number of open-arm entries (*p* = 0.42), **(C)** open-arm time (*p* = 0.26), or **(D)** total-arm entries (*p* = 0.47). **(E,F)** In the social approach test, late postpartum dams undergoing pup removal and postpartum social isolation (No Pups-ISO) exhibited **(D)** reduced social sniff time of a younger same-sex conspecific compared with the control dams (Pups) or dams that had pups removed but were pair-housed (No Pups-PH) (one-way ANOVA; *p* < 0.05) but no differences in total locomotor activity, as indexed by comparable numbers of total chamber crossings (*p* = 0.66). Error bars represent mean ± SEM. *p < 0.05.

Animals received a 1-h habituation to the testing room prior to testing. Behaviors were recorded and scored by an experimenter blind to the experimental condition. The FST and elevated plus maze (EPM) were conducted during the light cycle (11:00 a.m.–4:00 p.m.); the sucrose preference test (SPT) and the social approach test (SAT) were conducted shortly after the onset of the dark cycle (7:00–11:00 p.m.), in accordance with our prior study on postpartum rats (36). The three groups of dams (e.g., Pups: *n* = 8, No Pups-ISO: *n* = 8, and No Pups-PH: *n* = 9) were tested in either the SPT or the FST (Figure 1A, Experiment 1), but they did not undergo electrophysiological recordings given our prior work showing that swim stress reduces VTA population activity, the primary measure upon which we focus, in female rats (22). Another cohort of dams (Pups: *n* = 8, No Pups-ISO: *n* = 8, and No Pups-PH: *n* = 9) were tested in the EPM and SAT on PD 20–23, in that order, and underwent electrophysiological recordings (Figure 2A, Experiment 2). Dams with pups underwent behavioral testing while they were still with pups to control for possible confounding effects of pup weaning on maternal behavior and to be consistent with our previous study in which late postpartum rats were also kept with pups throughout behavioral testing (36). Finally, a cohort of nulliparous rats (randomly assigned to Virgin-PH or Virgin-ISO) were subjected to the SAT (PD 21) and FST (PD 23) (Virgin-PH: *n* = 6, Virgin-ISO: *n* = 6) or VTA electrophysiological recordings (Virgin-PH: *n* = 6, Virgin-ISO: *n* = 5) during PD 21–23 (Figure 4A, Experiment 3).

#### Sucrose Preference Test

Dams were exposed to two bottles (water, 1% sucrose solution) over a 28-h period within the home cage. Bottle location was switched 2–3× to prevent the formation of location preference and avoid neophobia during testing (37). Following a 4-h food and water deprivation, dams were given access to two bottles (water, 1% sucrose) for 1-h shortly after the onset of the dark cycle (7–7:30 p.m.) in a clean cage. Fluid intake was measured immediately post-test. Sucrose consumption was calculated as the difference in sucrose bottle weight (g). Sucrose preference was expressed as the percentage of sucrose relative to the water consumed: (grams sucrose consumed/total grams liquid consumed) × 100.

#### Elevated Plus Maze

The EPM is a plus-shaped apparatus (50 cm above the floor) consisting of two enclosed arms opposed by two open arms as previously described (36). The rats were placed on the central platform facing an open arm and movement was recorded for 5-min with a camera positioned overhead. The percentage of open-arm entries (open-arm entries/total entries × 100) and open-arm time (time in open arms/total time × 100), defined as front two paws and head in the arm, were used as indices of anxiety-like behavior; the total number of entries was used as an index of locomotor activity. The EPM was cleaned with 70% ethanol between animals.

#### Social Approach Test

A three-chambered apparatus made out of opaque, black Plexiglas was used to assess social approach behavior (36, 38). Dams were placed in a smaller center chamber adjacent to two other chambers, each containing a wire cage that allows the test rat to see and smell its content but prevents aggression/sexual behaviors (39). After a 5-min habituation period to the apparatus, a novel, younger, same-sex rat that had previously been habituated to the wire cage (1 × 15 min) was enclosed inside it and placed in a side chamber. An inanimate object (toy rat) was placed inside the other wire cage as a novel object control. The test rat was then allowed to explore the entire apparatus and the time spent sniffing the receptacle containing the social stimulus (social sniff time) and the total number of chamber crossings were recorded for 10 min. The SAT was cleaned with Quatricide between animals.

#### Forced Swim Test

The FST took place in a clear Plexiglas cylinder (50 cm high) filled with water (25 ± 1°C) up to 38 cm in which animals received a 15-min habituation (Day 1) followed by a 5-min test on the next day (Day 2) (22, 36). On Day 2, immobility behavior, defined as making only minor necessary movements to maintain head above water (40), and latency to first immobility bout (≥5 s) were measured. Water was changed between animals and rats were removed and dried before being placed back in the home cage. Although there has been some controversy regarding the interpretation of FST immobility (41, 42), we included this test to place our findings within the context of prior work regarding the behavioral effects of permanent pup removal on the dam, which reported increased FST immobility duration (17).

### Electrophysiological Recordings

#### Surgery

Single-unit extracellular recordings (22, 43) were performed 1–2 days post-behavioral testing. Only dams tested in the EPM/SAT were used for recordings, since our prior work suggests no impact of these tests on VTA activity in adult female rats (36, 38). Rats were anesthetized with 8% chloral hydrate (400 mg/kg, intraperitoneally), placed in a stereotaxic frame (Kopf, Tujunga, CA, United States), and maintained at 37°C using a temperature-controlled heating pad (Fine Science Tools, Foster City, CA, United States). Anesthesia level was monitored periodically (foot-pinch reflex) and adjusted by administering chloral hydrate as needed. After clearing the skull of skin and fascia, a burr hole was drilled in the region overlying the VTA [from bregma, anteroposterior (AP): −5.4 mm, mediolateral (ML): +0.6 mm] on the right side of the brain.

#### VTA Sampling

The DA neurons were sampled by making 6–9 vertical electrode passes (tracks), each separated by 0.2 mm, in a predetermined pattern spanning the antero-posterior and medio-lateral extent of the VTA [AP: 5.4–5.7 mm; ML: 0.6–1.0 mm from bregma, and dorsoventral (DV): 6.5–9.0 mm from dura] using glass electrodes filled with 2% Chicago Sky Blue (Sigma–Aldrich, St. Louis, MO, United States) dissolved in 2M saline that was lowered into the VTA using a hydraulic microdrive (Kopf). This procedure has been used by our group to sample DA neurons with a variety of different projection targets (44) in multiple studies (36, 45, 46). Signal was acquired using a preamplifier (Dagan, Minneapolis, MN, United States) and displayed on an oscilloscope (B&K Precision, Yorba Linda, CA, United States) with a signal fed to a computer running Lab Chart 7 (AD Instruments, San Diego, CA, United States).

#### Dopamine Neuron Identification

The DA neurons were identified with open filter settings (50 Hz low cuto, 16 kHz cutoff) using well-established electrophysiological criteria, including the location, slow, irregular firing pattern, long duration, variable shape biphasic action potential waveform (>2.2 ms), half width (>1.1 ms), and temporary cessation of firing during tail/foot pinch (47–49). Once identified, the DA neurons were recorded for 3 min (1-min minimum) when the signal-to-noise ratio exceeded 3:1. Three parameters of the DA neuron firing were measured: (i) number of spontaneously active DA cells per electrode track (i.e., CPT, also called population activity and refers to the number of active DA neurons found in each rat/the total number of tracks), (ii) basal firing rate, and (iii) proportion of spikes occurring in bursts, with burst initiation defined as the occurrence of two spikes with an interspike interval of ≤80 ms and the termination of burst defined as the occurrence of an interspike interval of >160 ms (50).

#### Placement Verification

Electrode placement was marked by electrophoretic ejection of Chicago Sky Blue dye at the final recording site. The rats were decapitated and the brains were removed and fixed in 8% paraformaldehyde for at least 48 h, transferred to 25% sucrose solution for cryoprotection, sectioned using a cryostat (Cryostar NX50, ThermoScientific, Waltham, MA, United States) into 60 μm coronal slides, mounted onto gelatin–chromalum-coated glass slides, and stained with cresyl violet and neutral red to check the recording electrode placement. Only animals with a minimum of six tracks within 0.4 mm of the target coordinates were included.

### Statistical Analyses

Behavioral data in postpartum females were analyzed using one-way analysis of variance (ANOVA) followed by Tukey’s multiple comparison *post-hoc* test when appropriate. Behavioral data in virgins was analyzed using unpaired *t*-tests. Electrophysiological data of the DA neurons were collected with Powerlab Lab Chart (AD Instruments) to identify spike time courses and exported to Neuroexplorer (NEX Technologies, NexTech Systems) software to calculate firing rate and burst firing. Track location data were analyzed by repeated measures (RM) two-way ANOVA. Electrophysiological data of three or more groups (postpartum females) were analyzed using one-way ANOVA or nested one-way ANOVA (firing rate, burst firing) followed by Tukey’s test when appropriate; pairwise comparisons (virgins) were analyzed using unpaired *t*-tests and nested *t*-tests (firing rate, burst firing). Correlation between social sniff time and active VTA DA cells (i.e., population activity) in postpartum females were assessed by Pearson’s r correlation analysis. The sample sizes were selected based on the only published study showing long-term behavioral alterations in dams following pup removal, and our prior study showed time-dependent changes in behavior and VTA activity during the postpartum (17, 36). Statistics were calculated using GraphPad Prism 9.0 and differences were considered significant when *p* < 0.05. Statistical outliers were identified using QuickCalcs Grubbs test (GraphPad) and excluded from the analysis.

## RESULTS

### Experiment 1: Pup Removal Has No Impact on Sucrose Consumption or Preference but Increases Passive FST Coping During the Late Postpartum Period

Dams were tested for sucrose consumption and preference on PD 21 (Figure 1A; *n* = 8–10 per group). No differences were found for sucrose consumption, as indexed by the difference in sucrose bottle weight measured in grams [one-way ANOVA: *F*_(2, 22)_ = 0.32, *p* = 0.73, Figure 1B]. No differences were found for sucrose preference, as indexed by a percentage of sucrose consumed [one-way ANOVA: *F*_(2, 22)_ = 1.99, *p* = 0.16, Figure 1C].

Dams were then tested in the FST on PD 23. A main effect of pup removal was found for time immobile [one-way ANOVA: *F*_(2,24)_ = 29.95, *p* < 0.0001, Figure 1D]. Compared with control dams (Pups: *n* = 8), both groups of dams experiencing pup removal (No-Pups-ISO: *n* = 9, No Pups-PH: *n* = 10) exhibited greater immobility duration (Tukey’s, *p* < 0.0001), and No Pups-ISO dams showed greater immobility than No Pups-PH dams (Tukey’s, *p* = 0.03; Pups: 13.88 ± 15.32 s, No Pups-ISO: 109.1 ± 24.35 s, No Pups-PH: 77.44 ± 32.38 s). A main effect of pup removal was found for latency to immobility [one-way ANOVA: *F*_(2,24)_ = 17.81, *p* < 0.0001, Figure 1E]. Compared with control dams with pups, both groups of dams experiencing pup removal exhibited reduced latencies to immobility (Tukey’s, Pups vs. No Pups-ISO: *p* < 0.0001, Pups vs. No Pups-PH: *p* = 0.0008; Pups: 186.1 ± 93.39 s, No Pups-ISO: 35.89 ± 20.84 s, No Pups-PH: 82.85 ± 34.05 s).

### Experiment 2.1: Pup Removal Spares Anxiety-Like Behavior but Reduces Social Motivation Only in Isolated Dams During the Late Postpartum Period

Anxiety-like and social approach behavior were tested in the EPM and the SAT, respectively, in a separate cohort of animals on PD 21 (Figure 2A; Pups: *n* = 8, No Pups-ISO: *n* = 8, No Pups-PH: *n* = 9). No effect was found for the percentage of open-arm entries [one-way ANOVA: *F*_(2, 22)_ = 0.88, *p* = 0.43, Figure 2B] or time spent in open arms [one-way ANOVA: *F*_(2, 22)_ = 1.42, *p* = 0.26, Figure 2C]. No differences in locomotor activity, as indexed by total-arm entries, were found [one-way ANOVA: *F*_(2, 22_) = 0.78, p = 0.47, Figure 2D].

A significant effect of pup removal was found for social approach [one-way ANOVA: *F*_(2,22)_ = 4.92, *p* = 0.02, Figure 2E]. Only dams that experienced pup removal and postpartum social isolation (No Pups-ISO: 112 ± 27.95 s) spent less time sniffing the social cage compared with the control dams (Pups: 164.5 ± 36.19 s; Tukey’s, *p* = 0.04) or dams that underwent pup removal and were PH (No Pups-PH: 166.3 ± 50.13 s; Tukey’s, *p* = 0.03). No between-group differences were found for locomotor activity [i.e., total number of chamber crossings; one-way ANOVA: *F*_(2,22)_ = 0.42, *p* = 0.66, Figure 2F].

### Experiment 2.2: Social Buffering of Long-Lasting Effects of Pup Removal on VTA DA Neuron Activity

**FIGURE 3 |:**
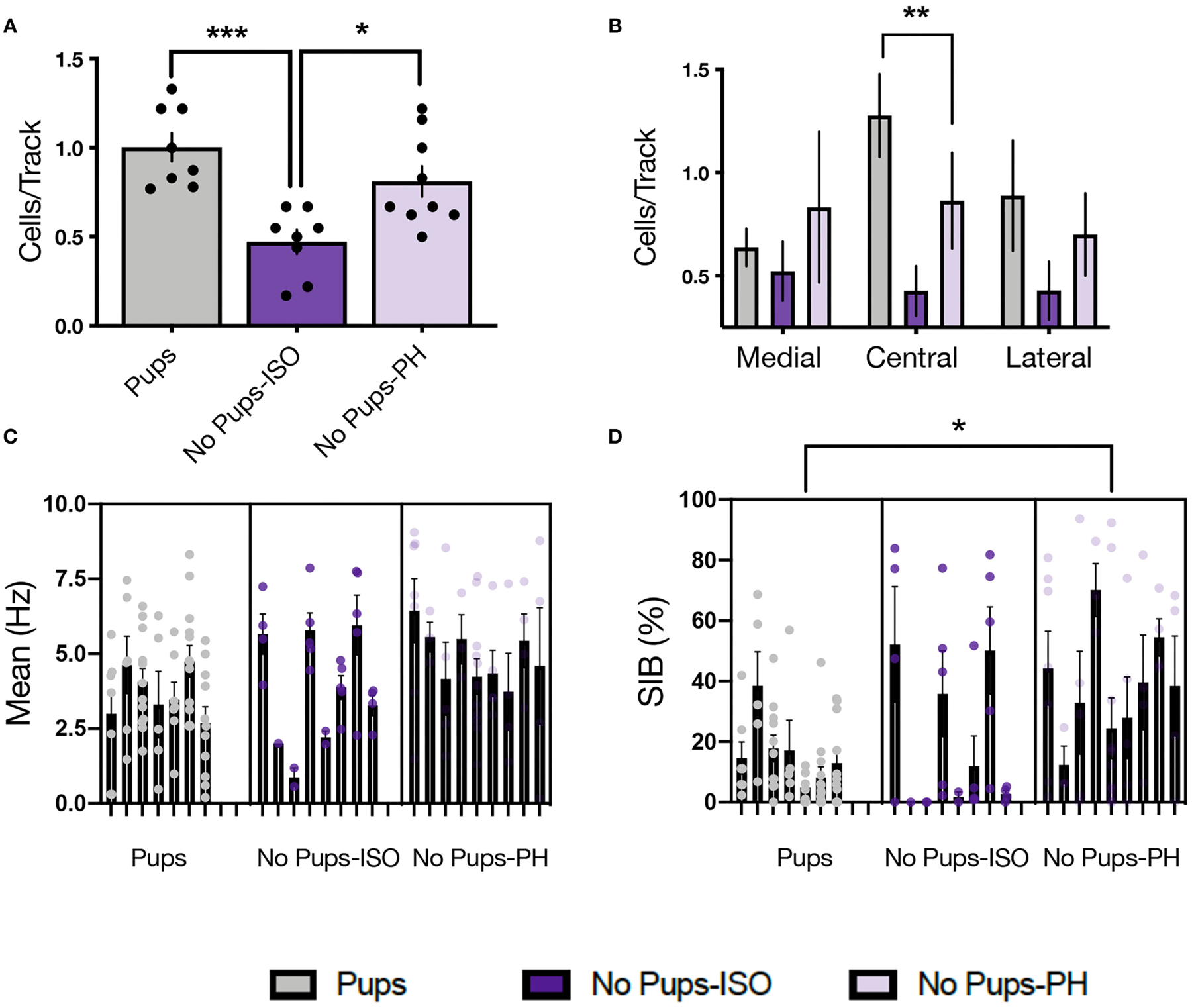
Pup removal resulted in long-lasting attenuation of ventral tegmental area (VTA) population activity only in dams that were isolated during the postpartum period. **(A)** A significant main effect of pup removal was found for VTA DA neuron activity in the late postpartum rats (one-way ANOVA; *p* < 0.001, *n* = 7–9 per group). Dams that underwent pup removal and postpartum isolation (No Pups-ISO) exhibited reduced numbers of spontaneously active DA neurons within the VTA compared with the control dams (Pups) or dams experiencing pup removal that were PH with another dam (No Pups-PH) (Tukey’s, *p* < 0.001 and *p* < 0.05, respectively). **(B)** No Pups-ISO dams exhibited a selective attenuation within the central tracks of the VTA (RM two-way ANOVA: *p* < 0.01). **(C)** No effect of pup removal was found on firing rate (nested one-way ANOVA; = 0.13). **(D)** A main effect was found on burst firing (nested one-way ANOVA; *p* < 0.05) in which No Pups-PH dams exhibited increased bursting activity (Tukey’s; *p* < 0.05) compared with controls (i.e., Pups). Error bars represent mean ± SEM. *p < 0.05, **p < 0.01, ***p < 0.001.

Single-unit recordings were conducted in dams 1–2 days after EPM/SAT, given our prior studies showed no impact of these tests on VTA activity in female and postpartum rats (36, 38) (Figure 2A; Pups: *n* = 8, 61 neurons; No Pups-ISO: *n* = 8, 28 neurons; No Pups-PH: *n* = 9, 46 neurons). The main effect of pup removal was found for CPT [i.e., active cells per track or population activity; one-way ANOVA: *F*_(2,22)_ = 11.33, *p* = 0.0004, Figure 3A]. Dams experiencing pup removal and postpartum social isolation exhibited lower numbers of active DA neurons within the VTA (i.e., attenuated VTA activity) (No Pups-ISO: 0.47 ± 0.19 CPT) compared with the controls (Pups: 1.00 ± 0.22 CPT; Tukey’s, *p* = 0.0003) and dams that underwent pup removal and pair-housing (No Pups-PH: 0.81 ± 0.26 CPT; Tukey’s, *p* = 0.01). No effect of pup removal on VTA population activity was observed in dams that were PH following pup removal, as they had similar numbers of active VTA DA neurons to control dams housed with pups (Pups vs. No Pups-PH, Tukey’s, *p* = 0.21). Pearson’s correlation analysis revealed a positive correlation between social sniff time in the SAT and active VTA cells/track (*r* = 0.72, *p* < 0.05) only in No Pups-ISO dams, suggesting a link between reduced social sniff time and blunted VTA activity in these animals. Moreover, the attenuation in VTA population activity observed in dams that underwent pup removal and isolation (No Pups-ISO) was driven by a selective reduction within the central tracks of the VTA [RM two-way ANOVA: *F*_(2, 15)_ = 4.21, *p* = 0.03, Figure 3B]. No differences were found for firing rate [nested one-way ANOVA: *F*_(2, 21)_ = 2.258, *p* = 0.13, Figure 3C]. A main effect was detected for burst firing [nested one-way ANOVA: *F*_(2, 21)_ = 4.317, *p* = 0.03; Figure 3D]. Dams that underwent pup removal and paired-housing exhibited an increased percentage of spikes in bursts compared with control dams with pups (Tukey’s, Pups vs. No Pups-PH, *p* = 0.02).

### Experiment 3: No Impact of Social Isolation on Social Motivation, Passive Coping, or VTA Activity in Adult Virgin Females

**FIGURE 4 |:**
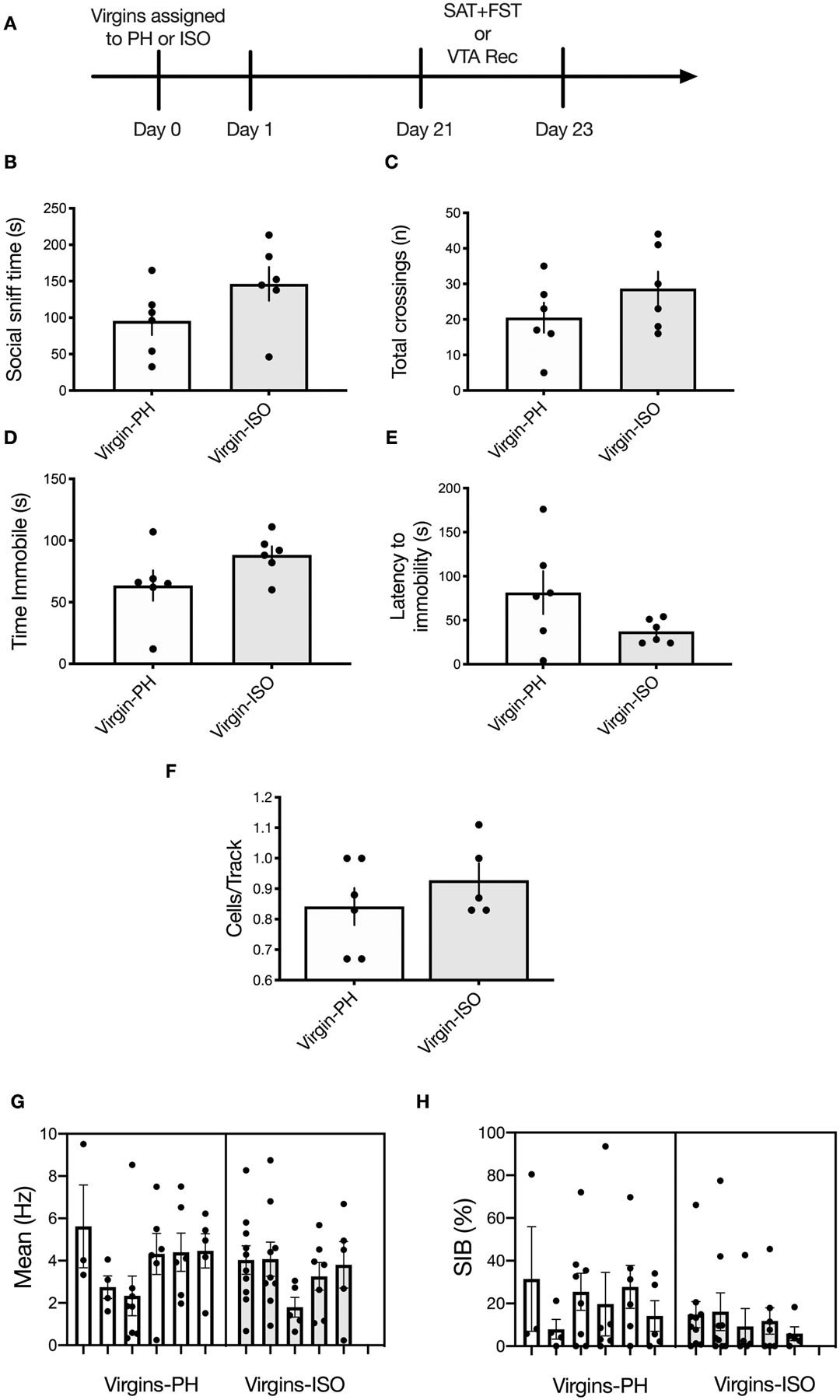
No impact of adult social isolation on behavior or VTA activity in virgin females. **(A)** Schematic of experimental timeline. Separate groups of rats were used for behavior and physiology. **(B,C)** No differences were found for **(B)** social sniff time (*p* = 0.12) or **(C)** total chamber crossings (*p* = 0.23) in virgins following 3-week isolation compared with the PH virgins (*n* = 6 per group, *t*-test). **(D,E)** Isolated virgins showed no differences in **(D)** time immobile (*p* = 0.11) or **(E)** latency to immobility during the FST (*p* = 0.11). **(F–H)** Social isolation did not impact any of the VTA activity parameters measured (*n* = 5–6 per group), as no between-group differences were found for **(F)** active cells per track (*p* = 0.33). **(G)** Firing rate (nested *t*-test: *p* = 0.62) or **(H)** percentage of spikes in bursts (nested *t*-test: *p* = 0.11). Error bars represent mean ± SEM.

To determine whether a 3-week isolation period (the same amount of time the dams used in experiments 1 and 2 were kept in their conditions) is sufficient to induce the neurobehavioral effects observed in No Pups-ISO dams, a separate cohort of virgins was subjected to 3 weeks of paired housing (Virgins-PH) or isolation (Virgins-ISO) and tested in the SAT, FST, or underwent electrophysiological recordings (Figure 4A). There was no difference in social sniff time (*t*_10_ = 1.694, *p* = 0.1211, *n* = 6; Figure 4B) or the number of chamber crossings (*t*_10_ = 1.277, *p* = 0.23; Figure 4C). No impact was found for FST immobility duration (*t*_10_ = 1.751, *p* = 0.11; Figure 4D) or latency to immobility (*t*_10_ = 1.769, *p* = 0.11; Figure 4E). In terms of VTA activity parameters (*n* = 5–6 animals per group, 32–36 neurons per group), there was no effect on cells/track (*t*_9_ = 1.033, *p* = 0.33), firing rate (*t*_9_ = 0.5146, *p* = 0.62), or burst firing (*t*_66_ = 1.635, *p* = 0.11; Figures 4F–H).

## DISCUSSION

This study tested the impact of pup removal on maternal affect and VTA activity during the late postpartum period, and whether these effects could be modulated by social support, as modeled by co-housing with an age- and experience-matched conspecific. We showed that pup removal induces long-lasting alterations in a subset of affect-related behaviors associated with depression-related phenotypes (passive coping, reduced social motivation) as well as blunted mesolimbic DA function (decreased VTA activity). These effects are specific to pup loss from PD 1 to 21, as 3-week social isolation had no impact on behavior or VTA activity in virgins. Furthermore, our results suggest that social buffering *via* housing with a conspecific can prevent pup-removal effects on a subset of these behaviors and VTA DA activity.

Pup removal had no effect on maternal sucrose consumption or preference, as both groups of dams experiencing pup removal exhibited comparable sucrose bottle weight and percentage of sucrose consumed as the controls. Our results differ from those reported earlier following repeated long-term maternal separations, which reported decreased intake of a 2.5% sucrose solution at PD 25 (14) and increased intake of a 10% solution 2–4 weeks after pup weaning (51). These data suggest that effects of pup separation on maternal sucrose consumption/preference are complex, and likely depend on the concentration of the solution, testing duration, strains used, as well as the timing of testing following pup deprivation. Our finding of no impact of pup removal on these measures may also suggest distinct effects of permanent pup removal vs. long- or short-term deprivations on the dam. For instance, the dams experiencing long-term pup separation (3 h × day, PD 2–14) exhibited reduced sucrose intake compared with the dams experiencing brief pup separations (15 min × day, PD 2–14) (14), and the dams experiencing a brief separation showed reduced sucrose consumption compared with dams kept with pups (52). Thus, both short- and long-term separations reduce sucrose consumption in the dam, but there was no impact of permanent pup removal on sucrose consumption in the late postpartum rats (current study) or the early postpartum rats (53).

Pup removal increased FST immobility duration and reduced latency to immobility. This is consistent with studies indicating that both permanent pup removal and long-term repeated maternal separations increase FST immobility in dams after weaning of pups (15, 17), and prior studies showing increased FST immobility in rodent models relevant to postpartum depression (PPD) (54–58). Moreover, we showed that pup-removal effects on the dam’s FST performance emerged prior to weaning and were resistant to social buffering, as both No Pups-ISO and No Pups-PH dams showed increased FST immobility and reduced latency to immobility compared with controls (Pups). However, No Pups-ISO and No Pups-PH dams spent different levels of time immobile compared to each other, which may suggest that increased FST immobility following pup removal can be partially ameliorated by pair-housing.

Pup removal had no impact on anxiety-related behaviors in the EPM. These data are in accordance with a prior study showing no differences in time spent in the open arm following pup removal compared with the dams with the pups (17), as well as earlier reports indicating no impact of long-term, repeated pup separations in the percentage of open-arm time or locomotion in an open field (11, 59). Interestingly, the behavioral effects of both pup removal and long-term pup separation are in contrast with those obtained following brief mother–pup separations, which have no impact in the FST (60) but increase anxiety-related behavior in the EPM (11). This suggests that pup removal, prolonged separations, and brief repeated separations exert distinct and dissociable effects on depression-like and anxiety-like behaviors in the dam. In this context, longer separations and permanent pup removal increase depression-like behavior in the FST but have no impact on anxiety-like behavior in the EPM, whereas brief and repeated pup separations have no effect on the FST immobility but increase anxiety-like behaviors in the EPM. Thus, the duration of the separation appears to determine which phenotype (i.e., anxiety- vs. depression-like) will emerge during the late postpartum period.

Pup removal attenuated social approach behavior during the late postpartum period, as indexed by less time spent sniffing a cage containing a younger same-sex conspecific. However, only dams that underwent pup removal and postpartum social isolation (No Pups-ISO) exhibited lower social sniff time, interpreted as reduced social motivation (39), suggesting that social buffering acts against the effects of pup removal on social motivation in the dam. Importantly, we think the changes in social approach observed in No Pups-ISO dams are specific to pup removal and the postpartum period, given that isolation has no impact on social behavior in rats older than 5 weeks of age (61), which is consistent with the known sensitive period in infancy/post-weaning for social isolation effects on adult behavior (62–65), and studies showing no impact of post-weaning isolation on the adult social behavior of female rats (66). In fact, these findings were confirmed in the present study, as adult virgin female rats that previously underwent a 3-week isolation period showed no alterations in social approach or FST coping compared with the pair-housed virgins. Collectively, our results suggest that dams require postpartum pup exposure to maintain appropriate levels of social approach and are consistent with our prior results showing attenuated social motivation in the early postpartum dams, which also exhibit passive FST coping (i.e., greater immobility) (36).

This is the first study showing that pup removal induces long-lasting alterations in the activity of VTA DA neurons *in vivo*. Dams that underwent pup removal and postpartum social isolation (No Pups-ISO) had lower numbers of spontaneously active VTA DA neurons compared with the control dams (Pups) and dams that had pups removed but were pair-housed (No Pups-PH). This reduction in VTA population activity was correlated with reduced social sniff time only in No Pups-ISO dams. Moreover, attenuation in active DA neurons was more pronounced within the central parts of the VTA, which is similar to our group’s findings following chronic and/or repeated stress exposure (46, 67). Thus, pup removal induces long-lasting attenuation of VTA DA activity (i.e., reduced numbers of active DA CPT) that overlaps with that observed in animal models relevant to depression (19–21, 68, 69). This is significant given that phasic responses are the behaviorally salient outputs of the DA system in response to reward-related stimuli (70), but only active (i.e., firing) neurons can be triggered to burst in responses to a stimulus (71). Therefore, the number of active neurons firing (i.e., CPT, also known as population activity) is an essential metric in measuring responsivity to reward, as tonic DA neuron population activity would be the “gain” or the level of amplification of the phasic signal [i.e., more DA neurons firing, the bigger the phasic response to stimuli and *vice versa* (72, 73)]. Conversely, a decrease in VTA DA neuron population activity would decrease stimulus-driven DA neuron responses, leading to diminished mesolimbic DA system activation (20, 21). Importantly, the VTA effects induced by pup removal appear to be specific to pup loss during the postpartum period, as no differences were found between pair-housed and socially isolated virgins in any parameter of VTA activity.

Finally, the effects of pup removal on VTA DA neuron activity were prevented by social buffering, as dams that were co-housed following pup removal (No Pups-PH) did not exhibit reduced numbers of active VTA DA neurons compared with the controls. Instead, these dams exhibited increased DA neuron bursting activity. Notably, DA neuron burst firing is thought to promote increases in synaptic DA and reflects the functionally relevant signal sent to postsynaptic sites (72) so that DA bursting is associated with a greater degree of DA release (74). Given that increases in burst firing were observed only in dams that underwent pup removal and paired housing (No Pups-PH), and increases in burst firing appear to compensate for changes (i.e., reductions) in the midbrain DA neuron activity (75), we proposed that these adaptations may reflect compensatory processes contributing to the resilience against stress-induced decreases in social motivation and VTA activity resulting from pup removal, although this remains to be determined.

In sum, pup removal exerts a long-lasting influence on a subset of affect-related behaviors (FST coping, social motivation) and VTA activity in the dam, suggesting that offspring exposure and aspects related to mother–infant attachment (i.e., sensory cues, lactation, pup contact) are important determinants for establishing subsequent maternal affect and mesolimbic DA system activity. We showed that deprivation of salient, species-expected social relationships (i.e., pups) precipitates an enduring negative affect state (i.e., passive coping and social anhedonia) and DA hypofunction (i.e., attenuated VTA activity) in the rat dam. Thus, maternal mood during postpartum is dependent on pup exposure, which appears to be important for the reduction in maternal depression-like behaviors as well as establishing proper social and mesolimbic DA function during the late postpartum period. Finally, we demonstrated that a subset of these effects (i.e., social motivation and VTA activity) induced by pup removal are prevented by/sensitive to social buffering. Collectively, these findings highlight the importance of social relationships during the postpartum period in female rats and that early pup removal may represent a novel model for studying adversity-induced changes in the maternal brain useful for the study of PPD as well as grief after the loss of a child. Future studies should be conducted to determine the persistence of pup-removal–induced deficits in social behavior and VTA function, including whether these extend into future pregnancies and influence subsequent maternal behaviors. Since the mesolimbic DA system provides a link between pup stimuli and rewarding events (25), long-lasting deficits in DA system function following pup removal could prevent or interfere with the normal association between pup and reinforcement, thereby disrupting mother–infant interactions and impairing motivated maternal behavior.

## Data Availability

The raw data supporting the conclusions of this article will be made available by the authors, without undue reservation.
